# Divergence Between Pain and Functional Recovery After Rotator Cuff Repair: A Prospective Comparative Analysis of Visual Analogue Scale, Constant-Murley Score, Disabilities of the Arm, Shoulder and Hand, and Shoulder Pain and Disability Index Scores

**DOI:** 10.7759/cureus.116304

**Published:** 2026-09-15

**Authors:** Sanket Mishra, Ananta N Panda, Shubhranshu Choudhary, Subham Panigrahy, Aklesh Panigrahy, Nikhil Ranjan

**Affiliations:** 1 Department of Orthopaedics, Institute of Medical Sciences and Sum Hospital, Bhubaneswar, IND

**Keywords:** constant murley score, correlation analysis, dash score, rotator cuff repair surgery, spadi score, supraspinatus tendon tear, vas

## Abstract

Introduction: Rotator cuff tears are a common injury, with patients presenting with shoulder pain and functional limitation. Clinically significant tears are usually managed by surgery, with the hope of faster and superior recovery, both in terms of pain relief and shoulder function. In day-to-day practice, we often use good pain relief as a sign that the patient is doing well overall. However, whether pain relief and functional recovery actually move together, or whether one lags behind the other, is not something that is usually checked directly in the same group of patients.

Objective: The aim of this study was to evaluate the trajectories of pain, measured by the visual analogue scale (VAS), and function, measured by the Constant-Murley Score (CMS), Disabilities of the Arm, Shoulder and Hand (DASH), and Shoulder Pain and Disability Index (SPADI), over the first year after rotator cuff repair, and to determine whether the extent of pain relief in a given patient corresponds to a matching improvement in function.

Methods: We carried out a prospective study on 40 patients who underwent surgery for a post-traumatic full-thickness supraspinatus tear at our institute, between February 2025 and July 2026. Pain and function were assessed using VAS, CMS, DASH and SPADI, recorded before surgery (pre-operatively), and again at 1, 3, 6 and 12 months after surgery. For every patient, the change in each of these four scores was calculated from the pre-operative reading to the 12-month reading. Pearson's correlation, calculated using Microsoft Excel (2024; Microsoft Corp., Redmond, WA, USA), was then used to see whether the change seen in VAS matched the change seen in CMS, DASH and SPADI in the same patient.

Results: The mean age of the patients was 40.6 ± 6.9 years, and the group included 26 men and 14 women. The mean VAS score improved from 7.85 ± 1.00 at baseline to 1.98 ± 1.14 at 12 months (a mean improvement of 5.88 points). The mean CMS improved from 62.83 ± 10.28 to 69.62 ± 3.81 (an improvement of 6.80 points). The mean DASH score improved from 71.47 ± 6.24 to 26.30 ± 5.71 (an improvement of 45.17 points), and the mean SPADI score improved from 65.20 ± 7.43 to 23.65 ± 10.58 (an improvement of 41.55 points). CMS showed an early fall at one month before improving by 12 months, whereas VAS, DASH and SPADI improved steadily from the start. The correlations between change in VAS and change in CMS, DASH, and SPADI were assessed using Pearson's correlation and were found to be r = -0.34 (p = 0.03), r = 0.38 (p = 0.01), and r = 0.18 (p = 0.27), respectively. The correlation between change in DASH and SPADI was r = 0.38 (p = 0.02). Overall, 33 of our 40 patients (82.5%) did not have any complications during the follow-up period.

Conclusion: Our study suggests that the amount of pain relief and the amount of functional gain do not necessarily match each other in the same patient after rotator cuff repair. A patient who reports good pain relief may not always show the best improvement in shoulder function, and the reverse may also be true. Pain score alone may therefore not give a complete picture of recovery. Recording a pain score together with a separate functional or disability score may provide a better assessment of postoperative recovery.

## Introduction

Rotator cuff tears are a fairly common problem that we come across in our day-to-day orthopaedic OPD, and may arise from a variety of mechanisms, including degenerative change, repetitive overuse, or acute trauma [1,2]. Post-traumatic tears, following a definite injury, are usually seen in younger and more active patients who put more demand on the shoulder. In such patients, surgery is generally advised, with the main aim of reducing pain and restoring the normal working ability of the shoulder [3]. The present study focuses specifically on this post-traumatic subgroup.

When we assess the outcome of this surgery, we generally rely on more than one type of score, since no single score can capture every aspect of recovery. The visual analogue scale (VAS) is used only for pain and is simple to record chairside [4]. The Constant-Murley Score (CMS) is assessed by the treating doctor and takes into account pain, movement of the shoulder, strength, and the ability to carry out daily activities [5,6]. Disabilities of the Arm, Shoulder and Hand (DASH), on the other hand, is a patient-reported outcome measure (PROM), that is, a questionnaire filled by the patient, and captures how much difficulty the patient feels while doing day-to-day and work-related activities of the upper limb as a whole [7]. Shoulder Pain and Disability Index (SPADI) is another PROM, a patient-filled questionnaire, but it is specific to the shoulder and looks at both pain and disability related to shoulder movements [8,9]. Unlike DASH and SPADI, CMS is not a PROM, since it is scored by the examining doctor rather than filled in by the patient. This is not just a difference in who fills out the form, but a real difference in what each score is actually measuring. CMS depends mainly on things the doctor checks directly on examination, like muscle strength and how far the shoulder can move, and these recover at the pace of tendon healing and muscle retraining. DASH and SPADI, on the other hand, depend on how the patient feels about their own pain and difficulty in daily tasks, which can be affected by things like pain tolerance, how the patient adjusts to the injury, and the patient's own expectations of recovery. Since these two types of scores are really looking at different things, one at physical healing and the other at the patient's own experience, it makes sense that they may not improve at the same pace in the same patient.

Since these scores assess different aspects of recovery, it is quite possible that a patient's pain settles down fairly quickly after surgery, while the shoulder itself continues to feel weak or difficult to use for daily chores for some more time. If we judge the success of surgery by pain relief alone, we may end up with an incomplete or even a misleading picture of how the patient is actually recovering. In our review of the literature, we found relatively few studies that had directly examined the relationship between pain improvement and functional improvement in the same group of patients.

With this background, we planned the present study to look at how pain (VAS) and function (CMS, DASH and SPADI) change over the first year following rotator cuff repair, and more importantly, to find out whether greater pain relief in a given patient is also accompanied by a correspondingly better functional recovery in the same patient.

## Materials and methods

Study design and setting

This was a prospective study conducted in the Department of Orthopaedic Surgery of our hospital, over a period from February 2025 to July 2026, and was carried out as part of a postgraduate dissertation. Approval for the study was obtained from our Institutional Ethics Committee before starting patient enrolment (IEC No. IEC/IMS.SH/SOA/2025/1115, dated 2 February 2025), and the study was conducted in keeping with the principles of the Declaration of Helsinki. Written informed consent was obtained from every patient before enrolment into the study.

It may be mentioned here that the study was not registered in a clinical trial registry prior to starting, since this was not a mandatory institutional requirement for this type of observational study. This has been stated as a limitation of the study.

Patients selection

Inclusion Criteria

We included patients between 20 and 65 years of age, of both genders, who gave a clear history of traumatic shoulder injury, and who had an MRI confirming a post-traumatic full-thickness tear of the supraspinatus tendon. The duration of injury was within eight weeks.

Exclusion Criteria

Patients with a chronic, degenerative type of tear without any history of injury, those who had undergone a previous surgery on the same shoulder, those having an associated fracture around the shoulder, those found to have an irreparable tear either on MRI or during surgery, or those with a duration of injury beyond eight weeks, making surgical repair difficult, were not included in the study.

There is no universal consensus in the literature on the exact cut-off duration separating an acute from a chronic rotator cuff tear, with reported thresholds ranging from as little as three weeks to as much as four months. Pogorzelski et al. suggested that repair of acute tears within eight weeks of injury may benefit from a superior biological healing environment, and we used this eight-week cut-off in our inclusion criteria on this basis [10].

In this way, 40 consecutive patients who fulfilled these criteria and gave their consent were enrolled in the study.

Surgery

Patients were randomly allocated in a 1:1 ratio for arthroscopic repair or open repair (mini-open repair) of the torn supraspinatus tendon. Randomisation was done by computer-generated block allocation, with the allocation concealed in sequentially numbered, sealed, opaque envelopes until the time of surgery. For the present analysis, patients from both groups were combined and analysed as a single cohort because the purpose of this study was to examine the relationship between pain and functional recovery, not to compare the two surgical techniques. All repairs, whether arthroscopic or mini-open, were performed using the same fixation construct: a double-row, suture-bridge (transosseous-equivalent) technique using two medial-row polyether ether ketone (PEEK) suture anchors (4.5 mm, Biotek VIMFIX-PK, 17 mm; Chetan Meditech Pvt. Ltd., Ahmedabad, India) and one lateral-row knotless anchor (5.5 mm, Biotek VIMFIX-LR, 19 mm; Chetan Meditech Pvt. Ltd.). As all patients were enrolled under a government-aided treatment scheme, the same implant system from a single vendor was used for all cases, ensuring uniformity of the fixation construct across the cohort.

Postoperative rehabilitation protocol

All patients followed a standardised four-phase postoperative rehabilitation protocol. In Phase 1 (0-6 weeks), the operated limb was kept in a sling continuously, day and night, for the first four weeks, followed by a period of partial sling use during weeks 5-6, with approximately three hours of daytime and two hours of night-time activity permitted out of the sling. Physiotherapy was introduced from the third week onward, comprising pendulum exercises, gentle passive range of motion, and stretching, with therapist-assisted passive range of motion added from weeks 4-5. No active lifting, pushing, or pulling was permitted during this phase. In Phase 2 (6-12 weeks), the sling was discontinued, and patients progressed to active-assisted and guided active range of motion exercises, gentle stretching, and light functional activities such as eating, drinking, writing, and mobile phone use, while heavy weight-bearing or resistive exercises remained restricted. In Phase 3 (12-18 weeks), patients began light resistance training, cuff stabilisation exercises, light isometrics, resistance band exercises, and painless active range of motion exercises. In Phase 4 (beyond 18 weeks), patients progressed to moderate and then heavy resistance exercises, with a gradual return to manual work, light sports, overhead activity, and driving. The same rehabilitation protocol was followed regardless of surgical technique (arthroscopic or mini-open).

Outcome scores

Pain was assessed using the VAS (scored 0 to 10) [4]. Function was assessed using the CMS (scored 0 to 100, with a higher score indicating better function) [5,6]. The CMS was assessed using a standard goniometer for the range-of-motion components. Strength was measured objectively using a digital dynamometer (Camry, 200 lb capacity; Zhongshan Camry Electronic Co., Ltd., Zhongshan, Guangdong, China (model EH101)). Disability was assessed using the DASH score [7] and the SPADI score [8,9], both scored from 0 to 100, with higher scores indicating greater disability. The VAS and CMS are established clinical assessment methods that are freely used in clinical practice. The DASH is copyrighted by the Institute for Work & Health and was used in its original, unmodified form for non-commercial research. SPADI is likewise non-proprietary and freely available [11], and was used in its original form. No modification was made to any of the instruments, and the original developers of each instrument have been credited through citation of their respective source publications. Published work has also examined the responsiveness of these scores after rotator cuff repair [12]. Published minimal clinically important difference (MCID) values are available for SPADI, CMS, DASH, and VAS and may help in interpreting the clinical importance of changes in these scores [13-16]. All four scores were recorded before surgery and at 1, 3, 6, and 12 months after surgery by an assessor who was not the operating surgeon.

Statistics

For every individual patient, we worked out the change in each of the four scores, from the pre-operative reading to the 12-month reading. For VAS, DASH and SPADI, since a fall in the score reflects improvement, change was calculated as (pre-operative value - 12-month value); for CMS, since a rise in the score reflects improvement, change was calculated as (12-month value - pre-operative value). With this convention, a positive change represents improvement for all four scores. The mean and standard deviation (SD) of this change were then found for each score. We also present the mean score at each time point, that is pre-operatively and at 1, 3, 6 and 12 months, so that the reader can see the pattern of recovery over the whole follow-up period, and not only the beginning and end values.

In order to find out whether the amount of pain relief matched the amount of functional recovery in these patients, we used Pearson's correlation (r) between the change in VAS and the change in each of CMS, DASH and SPADI. In the same manner, we also checked the correlation between the change in DASH and the change in SPADI, and between the change in CMS and the change in DASH/SPADI. A p-value of < 0.05 was considered to be statistically significant throughout this study. Microsoft Excel (2024; Microsoft Corp., Redmond, WA, USA) was used to make our calculations. Formal testing for normality of the distribution of change scores was not performed.

## Results

A total of 40 patients were included in our study, comprising 26 men and 14 women, with a mean age of 40.6 ± 6.9 years (range 30-50 years) and a mean BMI of 25.2 ± 1.9 kg/m². The tear was on the left side in 19 patients and on the right side in 21 patients. These baseline details are summarised in Table 1.

**Table 1:** Baseline details of patients (n=40).

Characteristic	Value (n=40)
Age (years), mean ± SD	40.6 ± 6.9 (range 30-50)
BMI (kg/m²), mean ± SD	25.2 ± 1.9
Sex	
Male	26 (65.0%)
Female	14 (35.0%)
Side	
Left	19 (47.5%)
Right	21 (52.5%)

Figure 1 shows intra-operative and clinical images of arthroscopic rotator cuff repair, including patient positioning and portal marking, the arthroscopic appearance of the tear before and after repair with a PEEK suture anchor, and clinical photographs showing the improvement in shoulder range of motion from before surgery to 12 months after surgery in a representative patient.

**Figure 1:**
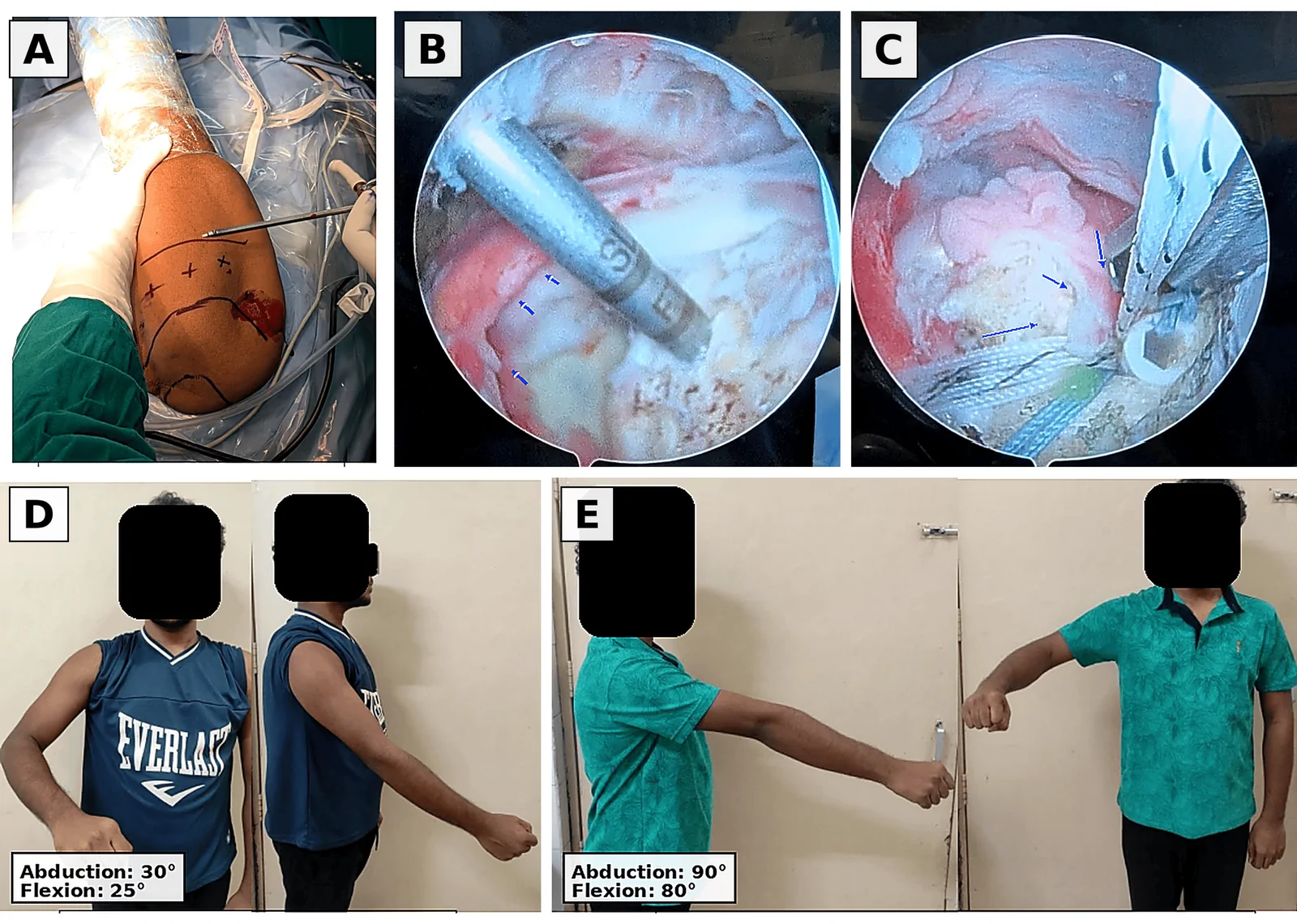
Intra-operative and clinical images of arthroscopic rotator cuff repair. (A) Patient positioning and arthroscopic portal marking prior to surgery. (B) Arthroscopic view of the supraspinatus tear, blue arrows indicating the torn tendon edge. (C) Arthroscopic view following repair using a polyether ether ketone (PEEK) suture anchor, blue arrows indicating the repaired tendon-bone interface. (D) Pre-operative shoulder range of motion: abduction 30°, flexion 25°. (E) Post-operative shoulder range of motion at 12 months: abduction 90°, flexion 80°. Reported range-of-motion values represent goniometer-measured clinical documentation; photographs are illustrative and may not precisely capture the exact end-range angle at the moment of capture.

Figure 2 similarly shows intra-operative and clinical images of mini-open rotator cuff repair. As both arthroscopic and mini-open repair were represented in this cohort, representative images of each technique are shown for completeness; these images are illustrative of the surgical and clinical assessment methods used in the study and are not intended as a comparison between the two techniques, which was outside the scope of this analysis.

**Figure 2:**
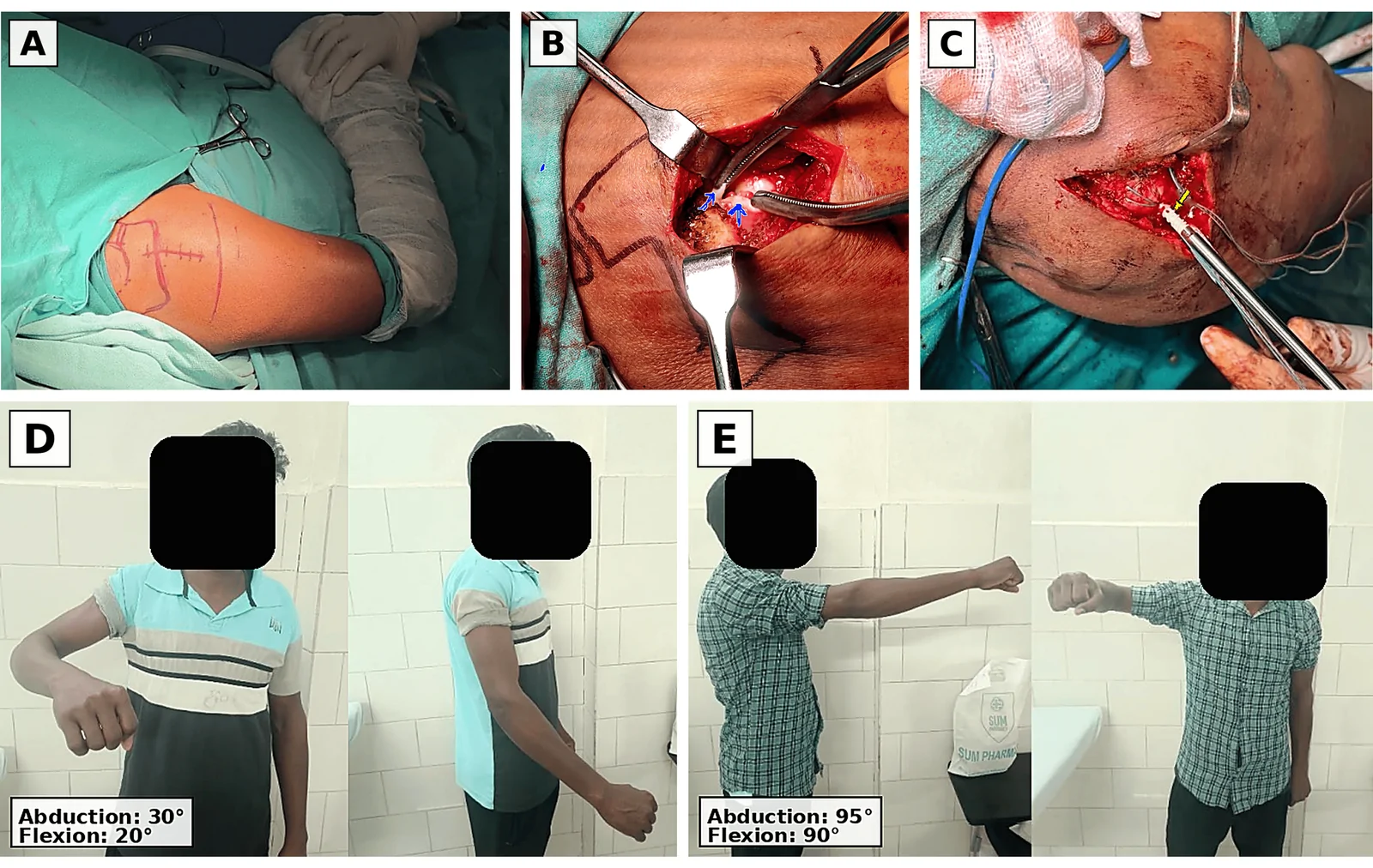
Intra-operative and clinical images of mini-open rotator cuff repair. (A) Patient positioning and skin marking prior to mini-open surgery. (B) Open surgical view of the supraspinatus tear, blue arrows indicating the torn tendon edge. (C) Open view following repair using a polyether ether ketone (PEEK) suture anchor, with a yellow arrow indicating the repaired tendon-bone interface. (D) Pre-operative shoulder range of motion: abduction 30°, flexion 20°. (E) Post-operative shoulder range of motion at 12 months: abduction 95°, flexion 90°. Reported range-of-motion values represent goniometer-measured clinical documentation; photographs are illustrative and may not precisely capture the exact end-range angle at the moment of capture.

Table 2 shows the mean score obtained for VAS, CMS, DASH and SPADI before surgery (pre-operatively), and at 1, 3, 6 and 12 months after surgery. VAS, DASH and SPADI all improved steadily from the pre-operative value onward. CMS, however, showed a different pattern: it actually fell below the pre-operative value in the first month after surgery, before climbing back up and eventually crossing the pre-operative value by around six months, and improving further by 12 months.

**Table 2:** Mean score (± SD) for VAS, CMS, DASH and SPADI before surgery (pre-op) and at 1, 3, 6 and 12 months after surgery (n=40). For VAS, DASH and SPADI, a lower score is better. For CMS, a higher score is better. VAS: visual analogue scale; CMS: Constant-Murley Score; DASH: Disabilities of the Arm, Shoulder and Hand; SPADI: Shoulder Pain and Disability Index

Score	Pre-op	1 month	3 months	6 months	12 months
VAS (pain)	7.85 ± 1.00	5.65 ± 0.77	4.05 ± 1.01	2.70 ± 1.11	1.98 ± 1.14
CMS (function)	62.83 ± 10.28	42.95 ± 3.23	53.48 ± 3.44	63.50 ± 3.73	69.62 ± 3.81
DASH (disability)	71.47 ± 6.24	60.48 ± 2.96	50.23 ± 3.48	40.48 ± 3.97	26.30 ± 5.71
SPADI (disability)	65.20 ± 7.43	51.08 ± 6.97	39.33 ± 7.24	29.82 ± 7.38	23.65 ± 10.58

Table 3 shows the mean change in each score from the pre-operative value to the value at 12 months. This gives a simple summary of the overall change during follow-up.

**Table 3:** Mean change (± SD) in VAS, CMS, DASH and SPADI, from the pre-operative value to 12 months (n=40). A positive change represents improvement for all four scores (a fall in score for VAS, DASH and SPADI; a rise in score for CMS). Note that the SD for CMS change is relatively large, reflecting the fact that CMS fell before it rose in several patients. VAS: visual analogue scale; CMS: Constant-Murley Score; DASH: Disabilities of the Arm, Shoulder and Hand; SPADI: Shoulder Pain and Disability Index

Score	Pre-op	12 months	Mean change
VAS (pain)	7.85	1.98	+5.88 ± 1.38
CMS (function)	62.83	69.62	+6.80 ± 11.96
DASH (disability)	71.47	26.30	+45.17 ± 7.57
SPADI (disability)	65.20	23.65	+41.55 ± 9.42

Table 4 shows the correlation between the change in VAS and the change in each functional score, from the pre-operative value to 12 months. The change in VAS had a weak to moderate negative, statistically significant correlation with CMS (r = -0.34, p = 0.03), which reflects the fact that CMS dipped before recovering, unlike VAS, which improved steadily from the start. The change in VAS showed a weak to moderate positive correlation with DASH (r = 0.38, p = 0.01), and a weak positive, non-significant correlation with SPADI (r = 0.18, p = 0.27). DASH and SPADI, both being patient-reported scores, showed a weak to moderate positive correlation with each other (r = 0.38, p = 0.02). A post-hoc subgroup analysis showed that the negative correlation between change in VAS and change in CMS was consistent in direction and similar in magnitude in both the arthroscopic (r = -0.39) and mini-open (r = -0.41) subgroups, suggesting that pooling the two techniques did not introduce a confounded or spurious result.

**Table 4:** Pearson's correlation (r) between the change in VAS and the change in CMS, DASH and SPADI, and between the functional scores, from the pre-operative value to 12 months (n=40). VAS: visual analogue scale; CMS: Constant-Murley Score; DASH: Disabilities of the Arm, Shoulder and Hand; SPADI: Shoulder Pain and Disability Index

Comparison (change, pre-op to 12 months)	r	p-value
VAS vs CMS	-0.34	0.03
VAS vs DASH	0.38	0.01
VAS vs SPADI	0.18	0.27
CMS vs DASH	-0.27	0.10
CMS vs SPADI	-0.11	0.51
DASH vs SPADI	0.38	0.02

Figure 3 shows scatter plots of these six relationships (panels A-F), with each point representing one patient. These plots visually support the correlation values reported in Table 4, illustrating the scatter and the weak-to-moderate trends described above, including the negative relationship between change in VAS and change in CMS.

**Figure 3:**
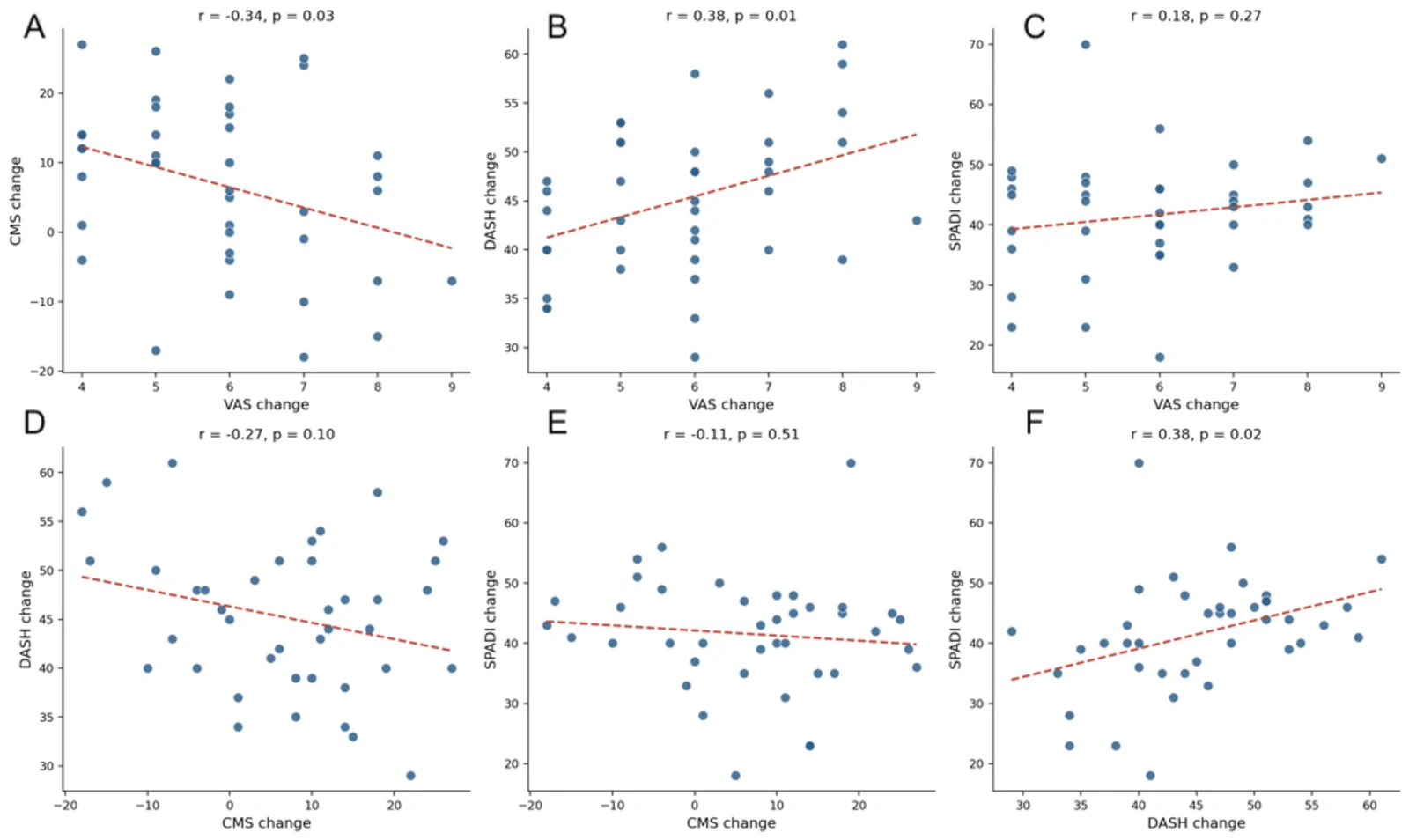
Scatter plots showing the relationship between change in VAS and change in CMS, DASH and SPADI, and between the functional scores themselves, from the pre-operative value to 12 months (n=40): (A) VAS-CMS; (B) VAS-DASH; (C) VAS-SPADI; (D) CMS-DASH; (E) CMS-SPADI; (F) DASH-SPADI. A positive value on each axis represents improvement. The dashed line in each panel is the line of best fit. VAS: visual analogue scale; CMS: Constant-Murley Score; DASH: Disabilities of the Arm, Shoulder and Hand; SPADI: Shoulder Pain and Disability Index

As far as complications were concerned, 33 of the 40 patients (82.5%) did not have any complications throughout the follow-up period. Among the remaining seven patients, five (12.5%) developed a re-tear (confirmed by ultrasonography), six (15.0%) had shoulder stiffness, and two (5.0%) had a superficial wound infection, with some patients experiencing more than one of these problems. It is worth mentioning that none of our patients required repeat surgery for these complications till preparation of this article and were managed conservatively.

## Discussion

The main finding to emerge from our study is that the amount of pain relief and the amount of functional recovery did not necessarily move together in the same patient following rotator cuff repair. We found that the change in pain, as measured by VAS, showed a weak to moderate positive correlation with the change in DASH, and a weak correlation with the change in SPADI. The change in VAS also showed a weak to moderate negative, statistically significant correlation with the change in the doctor-assessed functional score, CMS. On the other hand, DASH and SPADI, both being patient-reported measures, showed a weak to moderate positive correlation with each other, which we feel is a reassuring and consistent finding within our data.

The scatter evident in Figure 3 further illustrates this point. Even for the comparisons that reached statistical significance, such as VAS-CMS and DASH-SPADI, individual patients showed considerable spread around the line of best fit, meaning that pain relief in a given patient was, at best, a loose and imprecise indicator of the functional recovery achieved, and vice versa. This visual scatter reinforces our main finding: predicting an individual patient's functional outcome from their pain relief alone, or the reverse, would be unreliable, even though a weak-to-moderate trend could be seen at the group level across the cohort as a whole.

An interesting and somewhat unexpected finding of our study was the early behaviour of the CMS. While VAS, DASH and SPADI all improved steadily from the pre-operative value right through to 12 months, CMS actually fell below its pre-operative value in the first month after surgery, and only crossed back above the pre-operative value by around six months. We feel this is explained by the fact that CMS is not a pure pain score, but also includes range of movement and power, both of which are expected to be temporarily reduced in the early post-operative period, largely on account of the sling immobilisation, surgical-site pain, and stiffness that follow the surgery itself, quite apart from the original tear. This is consistent with our rehabilitation protocol, in which the shoulder remained in a sling continuously for the first four weeks, with only partial immobilisation and therapist-assisted passive motion introduced from the third to fourth week onward, and active-assisted motion and resistance training not begun until six and 12 weeks respectively. Since the one-month assessment therefore fell within a period of near-complete immobilisation, the dip seen in CMS at this time point is well explained by the rehabilitation timeline itself, rather than reflecting any adverse effect of the surgery. It should be noted, however, that this early dip explains the overall shape of the CMS recovery trajectory over time, but does not by itself account for the negative correlation observed between the net 12-month change in VAS and the net 12-month change in CMS (Table 4). This negative correlation instead reflects a more fundamental finding of this study: even when comparing pain relief and functional gain over the full follow-up period, the two did not necessarily move together within the same patient. This divergence between net pain relief and net functional gain is the central finding of our study, discussed further below.

A likely explanation for the overall pattern is that these various scores are, in a sense, looking at somewhat different aspects of the patient's recovery. VAS is purely a measure of pain, and pain may settle down fairly quickly as the initial post-operative inflammation, surgical-site pain, and stiffness subside. CMS, however, depends considerably on objective muscle strength and range of movement, both of which understandably take a longer time to return fully, as the shoulder gradually regains its strength and the patient slowly builds confidence in using the operated arm for regular activities. DASH and SPADI, on the other hand, both ask the patient directly about the difficulty faced while carrying out day-to-day and work-related tasks, and this shared way of assessing the patient's experience may well explain why these two scores moved somewhat together, even though neither of them correlated well with CMS.

Floor and ceiling effects may also have played a role in weakening these correlations. In our cohort, 11 of 40 patients (27.5%) had a VAS score of 1 or less by 12 months, including six patients who reported a VAS of 0. Once a patient's pain score reaches or nears this floor, further improvement cannot be captured numerically, even if the patient continues to recover in other respects. This restricts the spread of VAS change scores among our better-recovered patients, which can mechanically weaken any correlation between the change in VAS and the change in other scores, independent of whether a true underlying relationship exists. CMS, on the other hand, did not show a similar ceiling effect in our cohort (maximum CMS at 12 months was 76 out of a possible 100), largely because CMS includes a strength sub-component that tends to lag behind pain relief, since muscle strength takes longer to recover fully after a period of post-operative immobilisation and deconditioning. This uneven pace of recovery across the different components that make up CMS may further add to the weak correlation observed between VAS and CMS.

This pattern of recovery, with pain settling down comparatively early while doctor-assessed shoulder function actually dips before it improves, is also brought out by Table 2 of our study. This tells us that the timing of assessment can influence how complete the recovery appears to be, and that a single early postoperative assessment may not fully reflect the final functional recovery.

We feel this observation carries a useful message for routine clinical practice. A patient who reports feeling much better as far as pain is concerned may, at the same time, still be struggling with weakness or difficulty in performing daily activities with the operated shoulder, and this aspect could easily be overlooked if pain score alone is used to judge how well the patient has recovered. This is broadly in line with a recent study on shoulder PROMs after rotator cuff repair by Sahoo et al., which found that these scores mainly reflect a reduction in pain and better day-to-day function, and do not correlate well with actual structural healing of the tendon as seen on imaging [12]. Kurowicki et al. reported a similar pattern of pain relief outpacing functional recovery in a large cohort of patients undergoing arthroscopic rotator cuff repair, with around 75% of the eventual pain relief already achieved by three months, compared with only 45-58% of the eventual functional improvement at the same time point [17]. Published MCID values are also available separately for VAS, CMS, DASH and SPADI [13-16], but these MCID values, taken on their own, do not really tell us anything about how quickly each of these scores is expected to recover over time. It is also worth noting that although VAS, DASH, and SPADI all showed substantial improvement, the mean improvement in CMS (6.80 points) fell below published MCID thresholds for this score. This further illustrates that pain relief and patient-reported functional gain were not necessarily mirrored by an equivalent degree of objectively assessed functional improvement in our cohort, reinforcing the central finding of this study. Because of this, we feel that a single score should not be relied upon in isolation to judge the overall recovery of a patient after this surgery. Our findings would therefore support the practice of recording more than one type of outcome score together, that is, one score for pain, one that is assessed by the treating doctor, and one that is reported directly by the patient, when we evaluate outcome after rotator cuff repair.

It should also be noted that the patients had originally been allocated to two repair techniques for a different study as part of a postgraduate dissertation, but this particular study was not designed with the intention of comparing arthroscopic repair against open repair, and both these groups of patients were therefore combined into one cohort for the purpose of this analysis. Because of this, our findings should be taken as reflecting the general pattern of recovery seen after rotator cuff repair in our patient population as a whole, rather than as representing the effect of any one particular surgical technique. That said, the fact that a similar pattern of divergence between pain relief and functional recovery was seen across a mixed cohort of arthroscopic and open repairs, confirmed by a post-hoc subgroup analysis showing consistent VAS-CMS correlations in both groups (see Results), rather than being restricted to one technique, may also be viewed as a strength of this analysis, suggesting that this divergence is not specific to one particular surgical approach and could reflect a more general feature of recovery after rotator cuff repair.

Limitations

We would like to acknowledge a few limitations of our study. Firstly, this was a single-centre study involving 40 patients, and we feel that a larger, possibly multi-centric study would help provide a more precise and generalisable estimate of the correlation values that we have reported here. Secondly, the study was not registered in a trial registry prior to its commencement. Thirdly, since patients undergoing arthroscopic and open repair were combined into a single group for this analysis, we are unable to comment on whether this particular pattern of divergence between pain and function would differ between the two surgical techniques. Fourthly, the sample size was based on the number of eligible patients available during the study period and was not derived from a formal power calculation, which would ideally have been performed. In addition, formal normality testing of the outcome score distributions was not performed prior to selecting Pearson's correlation, which should be taken into account when interpreting the reported correlation coefficients and p-values. Lastly, it is important to remember that correlation, by its very nature, does not establish cause and effect; it merely tells us whether two measures tended to move together or not within the same patient, and should be interpreted with this understanding in mind.

## Conclusions

This study suggests that pain relief and functional recovery do not necessarily go hand in hand in the same patient after rotator cuff repair. A patient who reports good pain relief may not always show a matching improvement in shoulder function, and the reverse may also be true; pain and function appear to capture genuinely different, only loosely related, aspects of a patient's recovery, and the two can even move in different directions for some time after surgery.

This carries a practical message for both clinical practice and research reporting. Pain relief alone should not be taken as proof that a patient has also regained good functional use of the shoulder. We would recommend that a dedicated functional or disability score be recorded alongside the pain score, as a routine part of assessing and reporting outcomes after rotator cuff repair, so that a patient's recovery is captured more completely.
